# The Effortless Assessment of Risk States (EARS) Tool: An Interpersonal Approach to Mobile Sensing

**DOI:** 10.2196/10334

**Published:** 2018-08-28

**Authors:** Monika N Lind, Michelle L Byrne, Geordie Wicks, Alec M Smidt, Nicholas B Allen

**Affiliations:** ^1^ Center for Digital Mental Health Department of Psychology University of Oregon Eugene, OR United States

**Keywords:** passive mobile sensing, personal sensing, mobile sensing, mental health, risk assessment, crisis prevention, individual big data, telemedicine, mobile apps, cell phone, depression

## Abstract

**Background:**

To predict and prevent mental health crises, we must develop new approaches that can provide a dramatic advance in the effectiveness, timeliness, and scalability of our interventions. However, current methods of predicting mental health crises (eg, clinical monitoring, screening) usually fail on most, if not all, of these criteria. Luckily for us, 77% of Americans carry with them an unprecedented opportunity to detect risk states and provide precise life-saving interventions. Smartphones present an opportunity to empower individuals to leverage the data they generate through their normal phone use to predict and prevent mental health crises.

**Objective:**

To facilitate the collection of high-quality, passive mobile sensing data, we built the Effortless Assessment of Risk States (EARS) tool to enable the generation of predictive machine learning algorithms to solve previously intractable problems and identify risk states before they become crises.

**Methods:**

The EARS tool captures multiple indices of a person’s social and affective behavior via their naturalistic use of a smartphone. Although other mobile data collection tools exist, the EARS tool places a unique emphasis on capturing the content as well as the form of social communication on the phone. Signals collected include facial expressions, acoustic vocal quality, natural language use, physical activity, music choice, and geographical location. Critically, the EARS tool collects these data passively, with almost no burden on the user. We programmed the EARS tool in Java for the Android mobile platform. In building the EARS tool, we concentrated on two main considerations: (1) privacy and encryption and (2) phone use impact.

**Results:**

In a pilot study (N=24), participants tolerated the EARS tool well, reporting minimal burden. None of the participants who completed the study reported needing to use the provided battery packs. Current testing on a range of phones indicated that the tool consumed approximately 15% of the battery over a 16-hour period. Installation of the EARS tool caused minimal change in the user interface and user experience. Once installation is completed, the only difference the user notices is the custom keyboard.

**Conclusions:**

The EARS tool offers an innovative approach to passive mobile sensing by emphasizing the centrality of a person’s social life to their well-being. We built the EARS tool to power cutting-edge research, with the ultimate goal of leveraging individual big data to empower people and enhance mental health.

## Motivation

If health professionals cannot predict and prevent mental health crises, the field faces a crisis of its own. Although we have many evidence-based treatments, greater population-wide access to these treatments has thus far failed to yield significant reductions in the burden of mental health disorders [1-3]. This failure manifests, for example, in the meteoric rise of major depression in the World Health Organization’s rankings of conditions responsible for lost years of healthy life [4] and in the recent increase in suicide rates in some 50 World Health Organization member states, including an increase of 28% in the United States from 2000 to 2015 [5,6]. To predict and prevent mental health crises, we must develop new approaches that can provide a dramatic advance in the effectiveness, timeliness, and scalability of our interventions.

Intervening at critical moments in a person’s life—that is, during mental health crises, including times of risk for suicide, self-harm, psychotic breakdown, substance use relapse, and interpersonal loss—could dramatically enhance the effectiveness of mental health intervention. Even with the most extreme mental health crises, such as acute suicide risk, we find that the most effective interventions provide barriers to harmful behaviors *at the critical moment of action.* Take, for example, blister packaging on medications commonly used in suicide attempts. By placing a time-consuming, distracting barrier at just the right moment between a person and a suicide attempt, public health policy makers saved lives [7]. If we can improve the timeliness of our interventions, then even low-intensity interventions can have a major impact on improving health and saving lives [8]. Just-in-time adaptive interventions, delivered via mobile health apps and tailored to a person’s environment and internal state, headline a host of exciting developments in low-intensity, high-impact interventions [9].

Before we can take full advantage of these approaches, however, we face a critical challenge: *prediction*. It is one thing to recognize the tremendous power of precise timing for interventions, and it is quite another to possess the ability to identify the right time. Furthermore, as we face the challenge of predicting mental health crises, we must commit ourselves to meeting the highest standards of reliability, feasibility, scalability, and affordability. Given the large proportion of individuals experiencing mental health crises who do not receive treatment [10], prediction methods must reach those who are not in traditional mental health care. Current methods of predicting mental health crises (eg, clinical monitoring, screening) usually fail on most, if not all, of these criteria.

Luckily for us, 77% of Americans carry with them an unprecedented opportunity to detect risk states and provide precise life-saving interventions [11]. Smartphones enable the kind of access and insight into an individual’s behavior and mood that clinicians dream of. Packed with sensors and integral to many people’s lives, smartphones present an opportunity to empower individuals (and their clinicians, if individuals so choose) to leverage the data they generate every day through their normal phone use to predict and prevent mental health crises. This approach to data collection is called passive mobile sensing. Collecting high-quality, passive mobile sensing data will enable the generation of predictive machine learning algorithms to solve previously intractable problems and identify risk states before they become crises. To facilitate this goal, our team built the Effortless Assessment of Risk States (EARS) tool.

## Justification

We designed the EARS tool to capture multiple indices of behavior through an individual’s normal phone use. We selected these indices based on findings that demonstrate their links to mental health states such as depression and suicidality. These indices include physical activity, geolocation, sleep, phone use duration, music choice, facial expressions, acoustic vocal quality, and natural language use.

Physical activity, geolocation, sleep, phone use duration, and music choice data convey information about how individuals interact with their environments. Physical activity has a rich history of positive outcomes for mental health, including a finding from the Netherlands Mental Health Survey and Incidence Study (N=7076) that showed a strong correlation between more exercise during leisure time and both a lower incidence of mood and anxiety disorders and a faster recovery when those disorders do strike [12]. Geolocation overlaps with physical activity in part, but it can also provide important insight into the quality of a person’s daily movement and the environments in which they are spending their time. Saeb and colleagues demonstrated the power of 3 discrete movement quality variables derived from smartphone global positioning system (GPS) data to predict depressive symptom severity [13]. Meanwhile, environmental factors indexed by GPS data, such as living in a city and being exposed to green areas, have consequences for social stress processing and long-term mental health outcomes [14,15]. Sleep provides another powerful signal to help us predict depression and suicidality. One of the most common prodromal features of depression, sleep disturbance (including delayed sleep onset, difficulty staying asleep, and early morning wakening), also relates significantly to suicidality [16,17]. While evidence suggests that phone use duration may affect depression and suicidality via sleep disturbance [18], we believe that phone use duration also deserves study in its own right. For example, Thomée and colleagues found that high phone use predicted higher depressive symptoms, but not sleep disturbance, in women 1 year later [19]. Finally, music choice may provide affective insight, as recent findings suggest that listeners choose music to satisfy emotional needs, especially during periods of negative mood [20].

In contrast with the variables described above, EARS collects other signals that directly reflect both the form and content of an individual’s interpersonal engagement. Facial expressions can convey useful information about an individual’s mood states, an especially core feature of clinical depression. For example, Girard and colleagues found that participants with elevated depressive symptoms expressed fewer smiles and more signifiers of disgust [21]. Capturing these indicators of mood in a person’s facial expressions has become much more efficient and affordable in recent years with the advent of automated facial analysis [22].

While perhaps less intuitive than facial expression indicators, several aspects of acoustic vocal quality provide robust measures of depression and suicidality. These aspects include speech rate, vocal prosody, vowel space, and other machine learning-derived features [23-26]. Whereas acoustic voice quality ignores the semantic content of communication, natural language processing focuses on it. In addition to strong depression signals in written language in laboratory settings [27], accumulating evidence suggests that social media natural language content, especially expressions of anger and sadness, may identify suicidal individuals [28,29].

We are not the first research team to recognize the potential of passive mobile sensing to capture these behavioral indices. Numerous passive mobile sensing tools capture one or more of the variables described above. Table 1 [30-36] compares the 8 most feature-rich tools (Multimedia Appendix 1 compares the 12 most feature-rich tools [30-40]; for a broader survey of available tools, see the Wikipedia page “Mobile phone based sensing software” [41]).

Passive mobile sensing apps focus on sensor data (eg, GPS, accelerometer) and phone call and text messaging (short message service [SMS]) occurrence (eg, when calls and SMS happen, but not their contents). The EARS tool improves on these apps by adding several indices specifically relevant to interpersonal communication.

**Table 1:** A comparison of the most feature-rich, research-grade, passive mobile sensing apps^a^.

Feature	EARS^b^	AWARE [30]	EmotionSense [31]	Purple Robot [32]	Beiwe [33]	Funf [34]	RADAR-CNS^c^ [35]	StudentLife [36]
Geolocation	✔	✔	✔	✔	✔	✔	✔	✔
Accelerometer	✔	✔	✔	✔	✔	✔	✔	✔
Bluetooth colocation		✔	✔^d^	✔	✔	✔	✔	✔
Ambient light	✔	✔	✔	✔			✔	✔
Ambient noise		✔	✔	✔				✔
Charging time	✔							
Screen-on time	✔		✔		✔	✔		
App use	✔	✔	✔^d^	✔^d^		✔	✔	✔
Screen touch events					✔			
SMS^e^ frequency	✔	✔	✔	✔	✔	✔	✔	
SMS transcripts	✔			✔				
Call frequency	✔	✔	✔	✔	✔	✔	✔	✔
Browser history						✔		
In-call acoustic voice sample	✔							
All typed text	✔	✔						
Facial expressions	✔							
Music choice	✔							
Barometer		✔						
Wearables	✔						✔	
Video diary^f^	✔							
Audio diary^f^					✔			
Ecological momentary assessment^f^	✔	✔	✔		✔	✔		✔
Count	16	11	10	9	9	9	8	8

^a^EARS, AWARE, EmotionSense, Purple Robot, Beiwe, Funf, and RADAR-CNS are open source.

^b^EARS: Effortless Assessment of Risk States.

^c^RADAR-CNS: Remote Assessment of Disease and Relapse – Central Nervous System.

^d^Availability of the feature is limited to Android versions Kit-Kat 4.4 and below.

^e^SMS: short message service.

^f^Feature is not passive (ie, requires active engagement from the user).

## What the Effortless Assessment of Risk States Tool Does

The EARS tool captures multiple indices of a person’s social and affective behavior via their naturalistic use of a smartphone. As noted above, these indices include facial expressions, acoustic vocal quality, natural language use, physical activity, music choice, phone use duration, sleep, and geographical location. Critically, the EARS tool collects these data passively, with almost no burden on the user. For example, the EARS tool collects all language typed into the phone. These various data channels are encrypted and uploaded to a secure cloud server, then downloaded and decrypted in our laboratory. Preliminary analyses of these data are underway in our laboratory. Future iterations of the EARS tool will incorporate additional variables and automated analysis on the mobile device itself, which will facilitate both privacy and speed.

The first version of the EARS tool included four key features. A custom keyboard logged every *n* th word typed into the phone across all apps (*n* to be determined by the research team). A patch into the Google Fit application programming interface (Google Inc, Mountain View, CA, USA) collected physical activity data, including walking, running, biking, and car travel. A daily video diary used a persistent notification to prompt users to open the EARS app and record a 2-minute video of themselves talking about their day. While the video diary required active engagement of the user (similar to ecological momentary assessment), it provided a critical bridge in the early EARS tool while our team worked to develop passive means to capture facial expression and acoustic voice data. Each of the above data types (text entry, physical activity, and video diary) were tagged with the final data type of the first EARS tool: geolocation information. Since the Google Fit upload and video diary each occurred only once per day, the keyboard logger provided the richest source of geolocation data. Every time a user entered text into their phone, that text entry triggered a geotag. This approach to gathering geolocation data enabled us to avoid the battery drain of constant GPS data collection.

We piloted the first version of the EARS tool in the Effortless Assessment of Stressful Experiences (EASE) study (approved by the University of Oregon Institutional Review Board; protocol number 07212016.019). The EASE study employed an academic stress paradigm to test whether the EARS tool generates data that index stress. We recruited 24 undergraduate students over fall and winter terms of 2016 and 2017 via the Psychology and Linguistics Human Subjects Pool. We acquired informed consent (including descriptions of our double encryption and data storage procedure), then twice collected weeklong sets of passive mobile sensing data. We conducted the baseline assessment 3 to 7 weeks before the students’ first final examination (avoiding weeks when they had a midterm or other major project due) and the follow-up assessment during the week prior to the students’ last final examination. Self-report questionnaires of perceived stress and mental health symptoms were administered on the last day of each assessment and asked about symptoms over the past week. Participants tolerated the EARS tool well, reporting minimal burden. One individual declined participating in the study due to privacy concerns, and 1 individual declined due to dissatisfaction with the EARS keyboard. One participant dropped out due to privacy concerns, and 1 participant dropped out without explanation. None of the participants who completed the study reported needing to use the provided battery packs.

**Figure 1:**
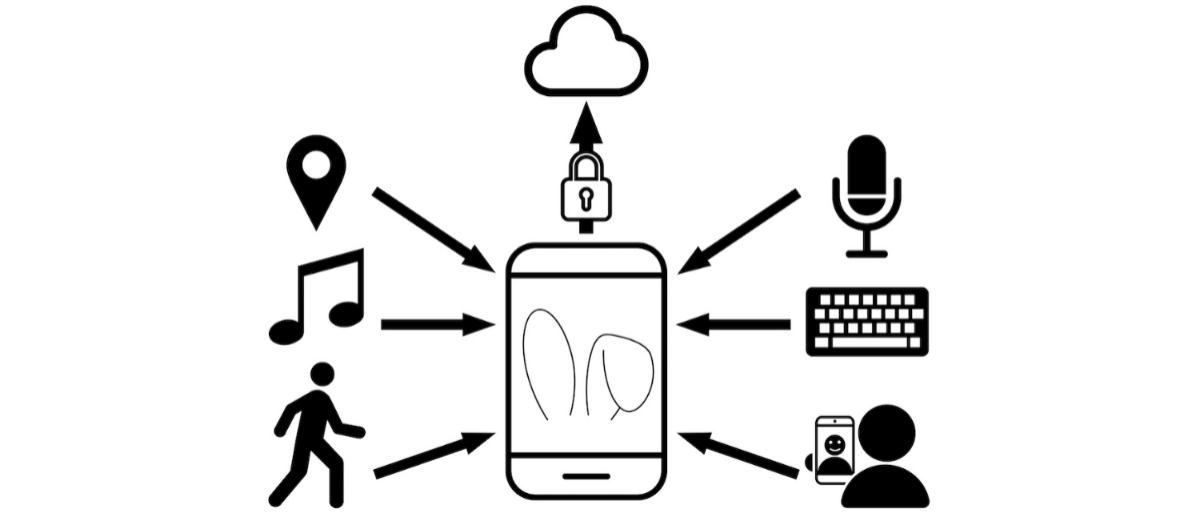
The Effortless Assessment of Risk States (EARS) tool collects multiple indices of behavior and mood.

The current version of the EARS tool includes the four features of the original, plus several enhancements (see Figure 1 [42-51]). The first major enhancement is the addition of a “selfie scraper,” which is a pipeline that gathers all photos captured by the device’s camera and encrypts and uploads them to our laboratory. During the decryption process, facial recognition software scans the photos and retains only selfies of the participant, discarding all photos that do not include the user’s face or that include other faces. This feature enables us to collect facial expression data passively, bringing us a step closer to full passivity. Second, the current version takes another step closer to full passivity with the addition of passive voice collection. The EARS tool records through the device’s microphone (but not the earpiece) during phone calls, encrypts these recordings, then uploads them for acoustic voice quality analysis. The third important upgrade of the current version is the constant collection of inertial measurement unit data. These data power fine-grained analysis of physical activity and sleep, over and above what we can glean from Google Fit data. Fourth, paired with the inertial measurement unit data, the ability to sample ambient light via the phone’s sensors further enhances the EARS tool’s measurement of sleep. The fifth new feature monitors the notification center to capture what music the phone user listens to across various music apps. Sixth, we have added the automatic collection of 4 indices of phone use: SMS frequency, call frequency, screen-on time, and app use time.

The current version of the tool also facilitates integration with wearable technology and adds customization for research teams. To facilitate integration of wearable technology, the EARS tool includes a mechanism to collect raw data from wearable devices (eg, wrist wearables that measure actigraphy and heart rate). This improves efficiency of data collection and reduces the burden on the participant because it cuts out the step of signing in to each participant’s individual wearable account to download these data. Capturing raw wearable data may also yield physiological variables that are otherwise obscured by the preprocessing that occurs in standard wearable application programming interfaces. One such variable is respiratory sinus arrhythmia, a measure of parasympathetic nervous system activity that is associated with emotional and mental health states [52].

We hope to share the EARS tool with other research teams, and we recognize that different research questions will call for different passive mobile sensing variables. As such, we are making the EARS tool as modular as possible, so that teams seeking to test hypotheses related to, say, natural language use and selfies do not also have to collect geolocation and app use data. By using Android’s flavors functionality, teams can compile a version of EARS that includes the code only for variables of interest, which helps to optimize the tool for battery drain and prevent unnecessary collection of confidential data.

A person’s smartphone use over time generates what we call “individual big data.” Individual big data comprise time-intensive, detail-rich data streams that capture the trends and idiosyncrasies of a person’s existence. With the EARS tool, we seek to harness these individual big data to power innovation and insight. Our research team aims to detect risk states within participants by determining a person’s behavioral set point and analyzing their deviations around that set point. We see this goal as one of many possible applications of the EARS tool.

## Effortless Assessment of Risk States Tool Engineering

We programmed the EARS tool in Java for the Android mobile platform (Google Inc). In building the EARS tool, we concentrated on two main considerations: (1) privacy and encryption, and (2) phone use impact. The EARS tool collects a massive amount of personal data, so ensuring that these data remain secure and cannot be used to identify users is of paramount concern. To achieve this, we have implemented a process to deidentify and encrypt the data.

To deidentify the data, the EARS tool uses the Android secure device identification (SSAID) to store and identify the data. The SSAID is accessible only when an Android user gives specific permission and is linked only to the hardware device, not a user or account name. We collect the SSAID and participant name at installation and store the SSAID key on non–cloud-based secure university servers. As a result, in the event of a breach of Amazon Web Services (AWS; Amazon.com, Inc, Seattle, WA, USA), there is no easy way for someone outside of our team to link the data with the name of the user who generated it. Obviously, this basic first step does not protect the actual content of the data. Therefore, we employ state-of-the-art encryption at multiple points in the pipeline.

After the sensors generate the data, the data are immediately encrypted by the EARS tool using 128-bit Advanced Encryption Standard (AES) encryption, a government standard endorsed by the US National Institute of Standards and Technology. On encryption, the unencrypted data are immediately deleted. When transmitting the data to AWS, the EARS tool uses a secure socket layer connection to the server, meaning that all data in transit are encrypted a second time using the industry standard for encrypting data travelling between networks. After transmission to the cloud, the EARS tool then deletes the encrypted data from the phone’s memory. On upload to AWS, the data are then also protected by Amazon’s server side encryption, which uses 256-bit AES encryption.

By encrypting the user data twice, once on the phone using our own encryption, and a second time on the AWS servers using Amazon’s, we can ensure that the data cannot be accessed at any time by anyone outside our team. This means that even Amazon does not have access to the unencrypted data, either by rogue employees or by government demands. We aimed to improve acceptability of the EARS tool for users by taking these steps to protect users’ data and allay privacy concerns.

We took another important step toward maximizing acceptability of the EARS tool by prioritizing phone use impact. The EARS tool runs in the background at all times. To minimize the impact on the user’s day-to-day experience, we have endeavored to make the EARS tool as lightweight as possible. First, the tool consumes around 30 MB of RAM. Most Android phones have between 2 and 4 GB of RAM, meaning the tool uses between 1% and 2% of memory. Second, the use of phone sensors can have a large impact on the battery life of a phone, as they draw relatively large amounts of power. To combat this, we have moved most cloud uploads to late at night when the phone is usually plugged in, and only when the device is connected to a Wi-Fi network. We also limit GPS readings to once every 5 minutes and, if possible, obtain location data from known Wi-Fi points, rather than connecting to a satellite. Current testing on a range of phones indicated that the tool consumes approximately 15% of the battery over a 16-hour period. Third, installation of the EARS tool causes minimal change in the user interface or user experience. Once installation is completed, the only difference the user will notice is the custom keyboard. With the exception of the optional video diary feature, everything else is collected in the background with no user interaction.

The EARS tool is hosted on GitHub (GitHub, Inc, San Francisco, CA, USA) on the University of Oregon, Center for Digital Mental Health’s page (GitHub username: C4DMH). It is licensed under the Apache 2.0 open source license, a permissive free software license that enables the free use, distribution, and modification of the EARS tool [53].

## Considerations

We must temper our enthusiasm for the exciting possibilities associated with these new types of data with careful consideration of several challenges. First, EARS is currently implemented only on the Android platform. While Android boasts 2 billion users worldwide, some 700 million people use an iPhone [54]. In the interest of eliminating as many sample selection confounding variables as possible, we have begun adapting the EARS tool for the iOS operating system. Given that most app developers begin on one platform, then port their product over to the other, EARS for iOS should match the functionality of EARS for Android; however, variation in software and sensors will likely result in some differences between the versions. The 4.6 billion people on this planet who do not use an Android or iOS smartphone present a much more stubborn challenge. That most of these people live in low- or middle-income countries or in countries outside of Europe and North America gives us pause, as we consider the historical affluent- and white-centric approach of clinical psychology. The steady increase in smartphone adoption around the world will probably reduce the impact of this limitation, but we remain mindful that the EARS tool carries built-in socioeconomic and cultural limitations alongside its passive mobile sensing features.

Those passive mobile sensing features generate significant ethical concerns as well, principal among which are recruitment and enrollment methods and protecting participants’ data. These concerns loom large in mobile health because they reflect the most prominent way in which for-profit app developers exploit smartphone users: app developers bury unsavory content in terms-of-service agreements they know users are unlikely to read, often leveraging these to sell users’ data. Some users are aware of these practices and may greet research using the EARS tool with skepticism. However, in our pilot study, only 2 of 28 screened participants declined further participation or dropped out due to privacy concerns. Nevertheless, this issue warrants attention as we scale up our studies and incorporate more diverse populations. The critical task, we believe, is to carefully assess who is empowered by the data and to clearly convey our priorities and precautions to the user. We value the empowerment of the user (eg, to take better control of their mental health) over the empowerment of a commercial or government entity (eg, acquiring user data without any benefit to the user). We oppose practices that empower others at the expense of the user and uphold respect for the user’s autonomy by insisting on an opt-in model for all applications of the EARS tool. This means that we reject any protocol that employs autoenrollment in its recruitment approach, such as embedding EARS features into an update of an existing app or requiring members of a specific health plan to participate. To date, we have acquired informed consent in person. As we scale our studies up and out, however, we will need to acquire informed consent remotely. To that end, we are developing a feature in the EARS tool to administer and confirm a participant’s informed consent.

Once a participant has opted in, our duty to protect their confidentiality and anonymity grows exponentially. The EARS tool encrypts all data locally on the phone as soon as the user generates them. Those encrypted data arrive in our laboratory via AWS, a US Health Insurance Portability and Accountability Act-compliant commercial cloud service that provides state-of-the-art security in transit. On completion of or withdrawal from a study, a participant’s uninstallation of the EARS tool automatically deletes all encrypted EARS data still residing on the phone. As a critical next step to ensure data privacy as we expand this line of research, we aim to conduct processing and analysis locally, on the participant’s phone, as soon as possible. In our current protocol, during a study, a participant’s encrypted data exist in 3 locations: the phone, the AWS cloud, and our laboratory’s secure server. These 3 storage locations increase the risk to the participant. As phones become more powerful and our research generates optimized data analysis algorithms, we aim to limit participants’ exposure to risk of privacy breach by executing our protocols within the phones themselves. We will take the first step in that direction soon—recent advances in facial recognition and automatic expression analysis software should enable us to locate the selfie scraper entirely on the phone so that only deidentified output will travel via the cloud to our laboratory [55].

## Future Directions

We have described in detail the data collection capabilities of the EARS tool. EARS data, however, are only as useful as the research questions they aim to answer. The next steps for our team include (1) testing the EARS tool with a large, representative sample to establish norms for each behavior, and (2) deploying the EARS tool in the next wave of an ongoing longitudinal study of adolescent girls. We are especially eager to collaborate with adolescent participants because they are digital natives, and we believe their data may be especially revealing. We aim to use the data from these 2 studies to determine which passive mobile sensing variables best predict mental health outcomes. As we discover the predictive power of each variable and the variables’ relationships with each other, we will optimize the data pipeline on the backend of the EARS tool to provide meaningful, manageable output for research teams.

Our team built this tool with the goal of predicting and preventing mental health crises such as suicide, but we believe that the EARS tool could serve many uses. The EARS tool could provide rich, observational data in efficacy and effectiveness studies in clinical psychology. It could also index general wellness via fine-grained data on activity, sleep, and mood for public health researchers. Furthermore, if we find that EARS data do predict changes in behavior and mood, medical researchers and health psychologists could use the EARS tool to derive mental and physical health insights. A future version of the EARS tool could provide clinical assessment and actionable feedback for users, including reports that a user could be encouraged to share with their primary care provider. We imagine exciting applications for social and personality psychologists to test self-report and laboratory findings against unobtrusive, ecologically valid behavioral data. Developmental psychologists, especially those who study adolescence, may also find that the EARS tool provides particularly rich insights, given adolescents’ extensive use of mobile computing and their status as digital natives. These applications of the EARS tool depend on rigorous signal processing, exploratory analysis, hypothesis testing, and machine learning methods. The potential is huge, and the work calls for the creative contributions of researchers from myriad areas of study.

## Conclusion

Engineers, mobile phone programmers, psychologists, and data scientists have done extraordinary work over the last decade, the sum of which could revolutionize mental health care. We believe that passive mobile sensing could be the catalyst of that revolution. The EARS tool offers an innovative approach to passive mobile sensing by emphasizing the centrality of a person’s social life to their well-being. We built the EARS tool to power cutting-edge research, with the ultimate goal of leveraging individual big data to empower people.

## Appendix

Multimedia Appendix 1 Comparison of the 12 most feature-rich, research-grade, passive mobile sensing apps.

## Abbreviations

AES: Advanced Encryption Standard
AWS: Amazon Web Services
EARS: Effortless Assessment of Risk States
EASE: Effortless Assessment of Stressful Experiences
GPS: global positioning system
RADAR-CNS: Remote Assessment of Disease and Relapse – Central Nervous System
SMS: short message service
SSAID: secure device identification
