# Supplementary material for: The Effortless Assessment of Risk States (EARS) Tool: An Interpersonal Approach to Mobile Sensing
Source: JMIR Ment Health. 2018 Aug 28;5(3):e10334. doi: 10.2196/10334 (PMC6134227; doi:10.2196/10334)
Supplement: Multimedia Appendix 1 [file mental_v5i3e10334_app1.pdf]

A comparison of the most feature-rich, research-grade, passive mobile sensing apps[a].

	EARS[b]	AWARE [30]	EmotionSense [31]	Purple Robot [32]	Beiwe [33]	Funf [34]	RADAR-CNS[c] [35]	StudentLife [36]	Cogito [37]	MoodScope [38]	MOSS[d] [39]	ResearchKit [40]
Geolocation	✔	✔	✔	✔	✔	✔	✔	✔	✔	✔	✔	✔
Accelerometer	✔	✔	✔	✔	✔	✔	✔	✔	✔		✔	✔
Bluetooth colocation		✔	✔[e]	✔	✔	✔	✔	✔				
Ambient light	✔	✔	✔	✔			✔	✔				
Ambient noise		✔	✔	✔				✔				✔
Charging time	✔											
Screen-on time	✔		✔		✔	✔			✔		✔	
App use	✔	✔	✔[e]	✔[e]		✔	✔	✔		✔		
Screen touch events					✔							
SMS[f] frequency	✔	✔	✔	✔	✔	✔	✔		✔	✔	✔	
SMS transcripts	✔			✔								
Call frequency	✔	✔	✔	✔	✔	✔	✔	✔	✔	✔	✔	
Email frequency										✔		
Browser history						✔				✔		
Calendar event frequency											✔	
In-call acoustic voice sample	✔											
All typed text	✔	✔										

Facial expressions	✔											
Music choice	✔											
Barometer		✔										✔
Wearables	✔						✔					✔
Video diary[g]	✔											
Audio diary[g]					✔				✔			
Ecological momentary assessment[g]	✔	✔	✔		✔	✔		✔	✔	✔		
Count	16	11	10	9	9	9	8	8	7	7	6	5

[a]EARS, AWARE, EmotionSense, Purple Robot, Beiwe, Funf, RADAR-CNS, and ResearchKit are open source.

[b]EARS: Effortless Assessment of Risk States.

[c]RADAR-CNS: Remote Assessment of Disease and Relapse – Central Nervous System.

[d]MOSS: Mobile Sensing and Support.

[e]Availability of the feature is limited to Android versions Kit-Kat [4.4] and below.

[f]SMS: short message service.

[g]Feature is not passive (ie, requires active engagement from the user).
